# Exploring the Use of Genomic and Routinely Collected Data: Narrative Literature Review and Interview Study

**DOI:** 10.2196/15739

**Published:** 2021-09-24

**Authors:** Helen Daniels, Kerina Helen Jones, Sharon Heys, David Vincent Ford

**Affiliations:** 1 Population Data Science, Swansea University Swansea United Kingdom

**Keywords:** genomic data, routine data, electronic health records, health data science, genome, data regulation, case study, eHealth

## Abstract

**Background:**

Advancing the use of genomic data with routinely collected health data holds great promise for health care and research. Increasing the use of these data is a high priority to understand and address the causes of disease.

**Objective:**

This study aims to provide an outline of the use of genomic data alongside routinely collected data in health research to date. As this field prepares to move forward, it is important to take stock of the current state of play in order to highlight new avenues for development, identify challenges, and ensure that adequate data governance models are in place for safe and socially acceptable progress.

**Methods:**

We conducted a literature review to draw information from past studies that have used genomic and routinely collected data and conducted interviews with individuals who use these data for health research. We collected data on the following: the rationale of using genomic data in conjunction with routinely collected data, types of genomic and routinely collected data used, data sources, project approvals, governance and access models, and challenges encountered.

**Results:**

The main purpose of using genomic and routinely collected data was to conduct genome-wide and phenome-wide association studies. Routine data sources included electronic health records, disease and death registries, health insurance systems, and deprivation indices. The types of genomic data included polygenic risk scores, single nucleotide polymorphisms, and measures of genetic activity, and biobanks generally provided these data. Although the literature search showed that biobanks released data to researchers, the case studies revealed a growing tendency for use within a data safe haven. Challenges of working with these data revolved around data collection, data storage, technical, and data privacy issues.

**Conclusions:**

Using genomic and routinely collected data holds great promise for progressing health research. Several challenges are involved, particularly in terms of privacy. Overcoming these barriers will ensure that the use of these data to progress health research can be exploited to its full potential.

## Introduction

### Background

The progression of genomics in the last few decades has been remarkable. Since 2001, when the Human Genome Project mapped and sequenced virtually every gene in the human genome, genetic sequencing technology has advanced rapidly in both the public and private domains. Next-generation sequencing costs have plummeted by almost 100%, and research opportunities have grown exponentially as a result [1]. For example, a simple search in the medical database, PubMed, shows that research on genomics has more than quadrupled since 2000, from around 340,000 published articles on this topic growing to 1.5 million by 2020. This increase has translated into quicker diagnoses, better outcomes, and more effective health care for patients [2-4]. Great strides have been made in cancer research, for example, where patients are now being treated according to their own or the tumor’s genomic data [5].

Being able to use genomic data in conjunction with routinely collected data holds even greater potential to advance knowledge by including factors wider in scope. Precision medicine requires that novel correlations of genotype, phenotype, and the environment be identified to inform new methods for diagnosing, treating, and preventing disease in a way that is responsive to the individual [5]. Knowledge of gene-environment interactions can also contribute on a population level by informing health and public health services in areas such as service planning, population genetic testing, disease prevention programs, and policy development [6]. Routinely collected data, electronic health records (EHRs) in particular, already hold vast amounts of clinical and environmental information on large numbers of people and preclude the need for lengthy and expensive data collection. Adding these phenotypic data to knowledge about a person’s genome can elucidate new knowledge, such as that about gene-environment and gene-drug interactions, and can thus provide a richer understanding of health and disease [7].

Increasing the use of genomic data for health research is a high government priority to understand and address the causes of disease. The potential of integrating genomic and routine data sets has been recognized in the United Kingdom by the Welsh Government [8] via their *Genomics for Precision Medicine Strategy* and by Genomics England [9] with the inception of the 100,000 genome project in 2018, both of whom are investigating ways to link genomic data and EHRs. From a more international perspective, the former president of the United States, Barack Obama, launched the Precision Medicine Initiative to improve individualized care by combining genomic data and EHRs with diet and lifestyle information from US citizens [10]. The UK Chief Medical Officer, Dame Sally Davies’s “genomic dream*”* of mainstreaming genomic medicine into National Health Service (NHS) standard care is becoming ever closer, which means that data from a person’s genome will likely be directly recorded into EHRs, making this type of research far more accessible [11].

### Objective

As this field prepares to move forward, it is important to take stock of the current state of play in order to highlight new avenues for development, identify challenges, and ensure that adequate data governance models are in place for safe and socially acceptable progress. Previous work has examined the benefits and logistical challenges of integrating genomic and routinely collected data in health care practice, but less is known of this specifically in a research setting [6,12,13]. Therefore, our objective is to add to this literature by conducting a narrative literature review and a series of interviews that would provide an outline of the use of genomic data alongside routinely collected data in health research to date. It focuses on the types of data that have been used, the role of routinely collected data in these studies, the data sources, how researchers access the data, and the challenges surrounding their use. This will inform further work in developing a framework for working with genomic and routinely collected data [14].

## Methods

### Literature Review

First, we conducted a literature search of research that used genomic data in conjunction with routinely collected data. We define routinely collected data as data collected as a matter of course and not specifically for research [8]. Genomic data refer to the data generated after processing a person’s genome, in full or in part, for example, by sequencing [7]. Studies were eligible for inclusion if they had used both types of data in combination to answer a health research question. We included studies of any design published in either peer-reviewed journals or gray literature in the English language.

We searched the following databases to identify these studies: PubMed, Ovid, CINAHL, OpenGrey, CENTRAL, LILACS, and Web of Knowledge from inception until January 31, 2019. We also searched for books, gray literature, and websites. We used a piloted search strategy that included keywords representing genetic and routinely collected data, and the following is the search strategy used for PubMed and modified for use with the remaining databases:

1. (Gene OR Genetic* OR Genome* OR Genomic*)
2. (Administration record* OR Anonymised OR Anonymized OR Anonymisation OR Anonymization OR *Big data* OR Clinical record* OR *Data linkage* OR *Data mining* OR *Data science* OR Education record* OR *Ehealth* OR EHR OR *Electronic data* OR Health record* OR Housing record* OR Encrypt* OR *Insurance* OR *Linked data* OR Medical record* OR Patient record* OR Prison record* OR *Publically available* OR *Publicly available* OR Register OR Registry OR Registries OR *Routine data* OR *Routinely collected* OR *Safe haven*)
3. #1 AND #2

This search in PubMed resulted in more than 50,000 hits. After initial pilot screenings, for example, by restricting to only publications within the last 10 years, it was clear that, given the number and heterogeneity of potentially relevant articles, neither a systematic review nor a meta-analysis would be practical. We took a pragmatic approach by scanning these articles to retrieve information on the following items until we reached data saturation, when no new information appeared in the text: types of genomic data; types and roles of routinely collected data; data sources; and data access models, that is, how researchers access the data. We chose examples from each criterion to ensure that we included a range of health conditions and presented them in a narrative format, which followed the format of the criteria given above.

### Interviews

To understand the use of genomic and routinely collected data in context, we recruited a purposive sample of individuals who have been involved in leading research projects using a combination of genomic and routinely collected data. We identified potential participants from the literature search outlined above and sent 19 interview invitations via email. Reminders were sent after 2 weeks, and if there was no response, we made no further contact. In total, 11 individuals agreed to participate in either an in-person or teleconferencing interview, depending on their geographical location. Participants were involved with the following projects: the Swansea Neurology Biobank (Swansea, Wales); Dementia Platform UK (Oxford, England); PsyCymru (Swansea, Wales); UK Biobank (Stockport, England); BC Generations Project (British Columbia, Canada); the Province of Ontario Neurodevelopmental Disorders (POND) Network, IC/ES (Ontario, Canada); Electronic and Medical Records and Genomics (eMERGE) Network (Vanderbilt, United States); and the Sax Institute’s 45 and Up study (New South Wales, Australia). We omitted any further information on the participants to maintain their anonymity. We developed interview questions with our advisory board: a group of UK geneticists and data scientists who were interested in using genomic data and our discussions centered around these:

1. What is the purpose of integrating the genetic data with health data?
2. What types of genetic data are being included?
3. Were there particular approvals you had to obtain? And if yes, what were these?
4. What were the main challenges encountered?
5. How did you address the challenges?
6. What is your main model for storing these data?
7. What access model(s) do you use? For example, safe room only, remote access, data released externally to researchers;
8. What are the conditions for access to data?

We followed up interviews by email if any answers needed clarification.

## Results

Textbox 1 provides a summary of the results of the literature review and interviews. Multimedia Appendix 1 Table S1 [15-32] provides a detailed summary of the results.

We included 19 studies in this literature review that provided broad examples of the different types of genomic and routinely collected data that can be used together to answer a health-related research question. The countries where this research was based were the United Kingdom [15-20], China [21], United States [22-29], Canada [19,30,31], and Australia [32].

**Summary of results.**

**Type of genomic data**

- Single nucleotide polymorphisms
- Polygenic risk scores
- Gene activity scores
- DNA methylation status

**Purpose of combining data**

- Genome-wide association studies
- Phenome-wide association studies
- Longitudinal studies
- Candidate gene studies
- Gene profiling studies
- Exploratory studies

**Types of routinely collected data**

- Electronic health records
- Disease registry data
- Disease registries
- Mortality registers
- Deprivation indices
- Health insurance

**Role of routinely collected data**

- Identifying cases and controls
- Baseline data
- Deep phenotyping
- Long-term follow-up
- Sociodemographic information

**Sources of data**

- Databanks
- Biobanks

**Governance models for data access**

- Publicly available on the web
- Released to researchers
- Data safe havens

**Challenges**

- Data collection
- Data storage and costs
- Technical and/or software issues
- Data privacy and data protection laws

### Types of Genomic Data

Examples found of the types of genomic data used in these studies included single nucleotide polymorphisms (SNPs), gene activity scores, and DNA methylation status. The most frequently used were SNPs [16,17,19,23-29], which represent a single base-pair change in the DNA sequence and are highly granular; hence, they are popular in health research [33]. Research studies tend to refer to SNPs by their reference SNP number, a unique identifier given to a SNP, or a cluster of SNPs by the National Center for Biotechnology (NCBI) [34].

Polygenic risk scores are closely related to SNPs and were used in 2 of the studies included in this review [15,16]. These predict a person’s likelihood of developing a particular disease based on the cumulative effect of a number of genetic variants [15]. Our literature search found other instances of quantitative measures of gene activity combined with routine data, including the 21-gene Recurrence Score, which measures the activity of 21 genes (16 cancer-related and 5 reference) for patients with breast cancer [33] and the enzyme activity score for the *CYP2D6* (cytochrome P450 family 2 subfamily D member 6) gene, which codes for an important drug-metabolizing enzyme, and is highly polymorphic in humans [34]. Rusiecki et al [29] measured changes in DNA methylation status in their participants to investigate whether this predicted posttraumatic stress disorder in US military service members.

### Purpose of Combining Genomic and Routine Data

Conducting genome-wide association studies (GWAS) [15,17-19,21], phenome-wide association studies (PheWAS) [24,25], and a combination of both [26,27] were the main purposes of combining genomic and routine data. Both these methods use powerful statistical techniques to find associations between genetic variants (SNPs) and phenotypes, which can then be used to predict the genetic risk factors of disease, levels of gene expression, and even social and behavioral characteristics such as educational attainment, impulsivity, and recreational drug experimentation [35,36]. Given the number of tested associations, these studies require large sample sizes to yield enough statistical power to detect differences in genetic variation between cases and controls [37]. Other study designs included longitudinal studies [16,18,30-32], a candidate gene study [23], case control studies [19,29], a gene profiling study [22], and an exploratory study [28]. Each of these studies was designed either to identify genetic risk factors of the disease or to investigate drug safety or efficacy (Multimedia Appendix 1 Table S1).

### Types and Role of Routinely Collected Data

EHRs appear to be, by far, the most common form of routinely collected data used in the studies identified in this review. For the type of research discussed here, data in EHRs have been used to identify eligible participants, for phenotyping, and to provide long-term follow-up on specific health outcomes. These data are collected as a matter of course in health care systems, and depending on their country of origin, EHR content can vary, although usually this digital record will include the patient’s name, address, demographics, medical history, care preferences, lifestyle information (such as diet, exercise, and smoking status), and free-text notes [38]. An example of EHR data used in the identified studies was the International Classification of Disease (ICD) coding. This allows clinicians to record the status of a patient in a standardized way, whether it is for disease, disorder, injury, infection, or symptoms [39]. Hebbring et al [26] used ICD codes in EHRs to identify appropriate cases and controls for their PheWAS study of the HLA-DRB1*150 gene involved in immune regulation. Rusiecki et al [29] used ICD codes to identify cases with a postdeployment diagnosis of posttraumatic stress disorder in their study on the link between gene expression and posttraumatic stress disorder in US military service members.

EHRs were also used to describe and validate a phenotype of interest, which is particularly important for PheWAS studies. The large number of phenotypes used in these analyses need to be clearly defined to ensure that any associations made with genetic variants are precise and replicable [40]. For example, Breitenstein et al [23] used EHRs to define the type 2 diabetes phenotype needed for their candidate gene study based on diagnoses, medications, and laboratory tests. The algorithm used to achieve this was developed by the eMERGE Network (see below for more details on this organization) and has been used successfully many times since [41].

In addition to providing a baseline snapshot of the patient, EHRs are also longitudinal in nature, and this makes them ideally placed to provide long-term follow-up to study participants. An example of this is the Genetics and Psychosis (GAP) study, which looked at a purported association between a variant of the *ZNF804A* gene and poor outcomes after first-episode psychosis [20]. Using individual-level linkage between EHRs and genotype data, this study followed the clinical outcomes for 291 patients over a period of 2 years, and subsequently found strong evidence for their hypothesis.

Other examples of routine data used in genomic research include disease registry morbidity and mortality records [20,22]. Disease registries collect information on clinical outcomes and care for a specific patient population over time. EHRs often feed data into these registries, but registry data can also include patient-reported outcomes and other biometric data, and therefore provide a more holistic view of the patient than an EHR would in isolation, for example, the UK MS Register [42]. Routine data sets need not be individual-level or person-based to prove useful in genetic research. A case in point is the Scottish Index of Multiple Deprivation, which gives an indication of a geographical area’s socioeconomic deprivation based on data about employment, income, health, education, housing, crime, and access to services [43]. Clarke et al [15] were able to link participants’ postcodes to the Scottish Index of Multiple Deprivation and generate socioeconomic deprivation variables to investigate their association with polygenic risk scores for alcohol dependence.

### Sources of Genomic and Routinely Collected Data

#### Overview

Although individual research projects often collect biological samples and generate their own genomic data for combination with routinely collected data [19,29], studies can also make use of the many sources of genomic and routinely collected data already available. Our interview participants spoke to us about the different data sources they used in their research and provided details on the use of these data and the participants of their projects. From this information, there seem to be two general categories of these sources: databanks and biobanks, the former where only the data are stored, and the latter, which store both biological samples and genomic data. Below are illustrative examples; therefore, this is not an exhaustive list.

#### Dementias Platform UK

Dementias Platform UK (DPUK) [16] is a data portal funded by the Medical Research Council that hosts the data of 2 million people from over 40 cohorts relevant to dementia research. Combining these data enhances the individual research power of each study and brings together knowledge from a number of stakeholders to facilitate and accelerate new discoveries. Examples of these cohorts are the GENetic Frontotemporal Dementia Initiative (GENFI; genotype data for *GRN*, *MAPT,* and *C9ORF72* genes) and the Genetic and Environmental Risk in Alzheimer’s Disease (GERAD) Consortium (whole exome sequences) [16]. Presently, DPUK provides EHR linkage for Welsh participants via the Secure Anonymized Information Linkage (SAIL) databank [44].

#### UK Biobank

UK Biobank [45] is a national resource with data from 500,000 participants aged between 40 and 69 years who have donated blood, urine, and saliva samples, have undergone a number of baseline measures, and have provided detailed health and behavioral information about themselves. Genomic data are available for 488,000 participants and comprise SNPs, genotypes, and haplotypes. The UK Biobank holds a number of routine data sets, including hospital inpatient episodes, cancer registrations, and deaths. Studies using the UK Biobank genomic and routinely collected data include genome-wide meta-analyses of depression [17] and identifying candidate gene and disease associations that could help predict adverse drug reactions [26].

#### Personal Genome Project

The Personal Genomes Project (PGP) is a databank founded in 2005 at Harvard University and now extends worldwide. It provides a web-based platform for individuals (over 100,000 people to date) to share their genomic data publicly, along with their EHRs, and other trait information to progress science without many of the governance restrictions of traditional research. Most of the genomic data on the PGP database are in SNP format, although files of raw sequence data are available for some participants [43].

#### eMERGE Network

The eMERGE Network is a consortium of American medical institutions whose goal is to use EHR data in combination with a variety of genomic data types to advance translational research. The network also releases its genomic data, including GWAS, whole genome, and whole exome sequence data, along with a subset of phenotypic elements to the broader community of researchers via the dbGaP—an NCBI [46] database of genotypes and phenotypes. Through this mechanism, any research project can be applied to the uploaded data. A wealth of publications have resulted from eMERGE’s work; these are available to view on the web [24,25,47].

#### POND Network

The POND network is an IC/ES [31] initiative based in Ontario, Canada, and involves a cohort of children and young people (the total number of the sample is approximately 3000) with a neurological development disorder, with a particular focus on autism. This is a highly phenotyped cohort with all participants having undergone multiple clinical tests, including those for attention-deficit/hyperactivity disorder, obsessive-compulsive disorder, and family history and demographic data. A subset (n=667) provided consent for linkage with administrative data held in IC/ES via health card numbers. The aim is to identify subgroups of autism based on the co-occurrence of other developmental conditions, other comorbidities, and health service use, and to characterize these groups based on clinical attributes and genomics. The network data are project-level only, although it is anticipated that linkage of genomic data to administrative and health data will become more routine in the future.

#### BC Generations Project

The BC Generations Project [48] is British Columbia’s (BC) largest ever health study and is part of a national initiative—the Canadian Partnership for Tomorrow Project—to aid researchers in answering questions about how environment, lifestyle, and genes contribute to cancer and other chronic diseases. Almost 30,000 participants were involved in the project, and they provided baseline information about their health, diet, lifestyle, and medical and family history. Many have also donated blood and urine samples, and the type of genomic data generated from these samples is based on the needs of the researchers (subject to approval). The BC cohort is one of several provincial cohorts that can be combined for national studies or can be linked to provincial administrative data via PopDataBC [49].

#### Sax Institute and the 45 and Up Study

Based in Australia, the main business of the Sax Institute is to manage the *45 and up* study, which has been following 260,000 people for over 12 years who provide both routinely collected and self-reported health data [32]. The aim of this study was to collect samples from 50,000 individuals for full genome sequencing. The Sax Institute has a partnership with the Garvan Institute of Medical Research [50], which acts as a genome sequencing facility and makes data available for research subject to approval. The Garvan Institute retains all the genomic data, but with data linkage to the Sax Institute.

### Data Governance Access Models

#### Overview

We surveyed the data access models and information governance systems of genomic and routine data sources identified by our literature search and interviews. From the additional information given to us by our interview participants, we were able to categorize data access models as follows: (1) publicly available on the web, (2) released to researchers, or accessed via a (3) data safe haven.

#### Publicly Available on the Web

There are a wealth of free, genomic data sources on the web, made available because of individual projects or from the pooled results of a variety of different projects. These data are either downloadable or viewable on the web. For this type of resource, data tend to be at the gene or variant level (eg, GIANT Consortium [51]; GWAS Catalog [52]), but some do hold individual-level data, eg, Database of Genomic Variants [53]. The Personal Genome Project, described above, is the only data source that provides identifiable, individual-level genomic data [43]. All other data were deidentified.

#### Released to Researchers

Currently, the most common way for genomic data to be accessed for research is through secure electronic file transfer to the researcher. This generally occurs after successful application to an internal review board (IRB) and signed data use agreement (DUA). All of the published research studies identified by our literature search used this model, unless the genomic data were generated specifically for that particular project. For instance, Cronin et al [24] received genomic data regarding 54 SNPs, and also demographic, vital sign, and billing data derived linked EHRs by the eMERGE network. Other biobanks using this model include Mayo Genome Consortia [23], China Kadoorie Biobank [21], UK Biobank [17,26], BioVu [24], and Generation Scotland [17], although access to BioVu data is only granted to Vanderbilt faculty members [54]. In addition to IRB and DUA procedures, the use of individual-level EHR data supplied by Generation Scotland also requires an application to the NHS Research Ethics Committee [55].

#### Data Safe Havens

The use of large-scale population data in health research has become increasingly popular in the last few decades, and this has seen the evolution of data safe havens as a way to ensure its safe and secure use. Lea et al [56] defined data safe havens as a system that invokes procedural, technical, and physical controls, including access to data within a secure environment (rather than data release), in order to safeguard the identities of people providing the data. Despite there being a greater tendency in the literature for releasing data to researchers, we have seen from our work with interview participants, a trend toward using a data safe haven system for both younger and more well-established organizations.

The PsyCymru study [18] and the Swansea Neurology Biobank [19] have deposited polygenic risk scores and SNPs, respectively, into the SAIL Databank [44], a data safe haven based at Swansea University. The SAIL Databank provides remote access to many linkable anonymized data sets, and both of these studies have used SAIL to link their genetic data and other phenotypic data to EHRs and other routinely collected data. These data are available only for project access, and currently cannot be shared. However, their intention is to make linked genetic and routinely collected data available for research in the near future [57,58].

The Sax Institute operates in a similar way to the SAIL Databank in that it provides access to genomic and health data via a virtual lab by remote access anywhere in the world. Data access requires approval by two data access committees: one at the Garvan Institute and one at the Sax Institute. The BC Generations Project said, “should the researcher access the project’s data via PopDataBC then they would only be allowed to use the data on within the secured research environment” (Participant 5, BC Generations Project).

For the UK Biobank, the current *modus operandi* releases anonymized data externally to researchers. However, they stated that this is unlikely to be sustainable because genomic data files are too large. The UK Biobank will likely be changing to a remote access model in the near future. The eMERGE Network also confirmed that they are experimenting with remote access into a data safe haven. One of our participants who used IC/ES data explained that “as the data are considered highly sensitive, access is only by an ICES analyst, with results provided to the project lead” (Participant 4, IC/ES).

### Challenges

Combining genomic and routine data does not come without its challenges. Our participants, who are currently working with these data, spoke to us about these different challenges during their interviews, and their experiences are summarized below.

#### Data Collection

A challenge described by several participants concerned data collection. Conducting long-term follow-up over long periods and at regular intervals means that participants need to be invited and reconsented to provide more blood and other health information. In addition, poor quality sequence alignment could render the samples useless, and with repeated use by researchers, blood samples will eventually become exhausted. Each of these issues necessitates a lengthy and expensive process of resampling thousands of individuals and imposes a burden on participants. A possible solution to this, as one participant suggested, is to “join up with others biobanks in order to work towards epidemiologically valid sample sizes” (Participant 1, 45 and Up Study)

#### Data Storage and Costs

Genomic data are huge, approximately 90 GB for the raw data of 1 whole genome, and this is often the source of technical issues surrounding its use [59]. One participant told us that their main challenge was data storage capacity and that “it may no longer be cost or space efficient for organisations to hold multiple datasets” (Participant 2, UK Biobank). They go on to explain that there is certainly a case to be made for storing genomic data as VCF files only, which keep record only of gene sequence variations and are much smaller and easier to work with. However, this restricts the type of analyses possible, particularly in the advent of new discoveries about the anatomy of the genome. There may be a need to find solutions regarding storage space for raw genomic data and for specialist platforms required to conduct analyses.

#### Technical and/or Software Issues

Many of the challenges faced by participants working with these data are technical in nature. Participant 3 (eMERGE) described creating a sequencing platform from the ground up, which was much longer than initially projected. Furthermore, they described that multiple sequencing centers needed harmonization, as well as the needs across sites and projects for network-wide data collection. Another participant found difficulties surrounding analysis software and analyst capacity, since this is a very specialized skill (Participant 4, IC/ES). Researchers often conduct genetic data analysis using publicly available, downloadable software applications, which are subject to frequent updates for improvements. This is a challenge to incorporate this software and keep it up to date. Several other participants spoke of similar software issues. Potential solutions discussed during our interviews were to upscale and have dedicated servers for genomic data, as well as to install specialist toolsets within the secure research environment.

#### Data Privacy and Data Protection Laws

Given the possibility of identifying individuals from genomic data [60], privacy is of primary concern. We were told that, for one research project using IC/ES data (Participant 4), the privacy approval group was concerned about identifiability due to the genomic data being unique, particularly where there are rare variants. This means that the genomic data contained in variant call files have been brought into the databank to show the feasibility of transfer but has to be integrated into the analytical platform.

The General Data Protection Regulation [61] in the United Kingdom states that if researchers are to rely on the lawful basis of consent to process medical genomic data, the reasons for doing so must be described in an explicit and transparent way. The rapid decrease in the cost of whole genome sequencing in the last few decades has opened many new avenues for genetic research, but this means that it is impossible to predict what this research will actually look like in the future [55]. One participant felt that this means they would need to seek consent from the participant for each new research proposal [62], rather than just once when their sample was taken. They expressed concerns that “continued and lengthy re-contact with participants was not only costly and difficult, it may also be invasive and burdensome” (Participant 11, Swansea Neurology Biobank). Also mentioned was Canada’s antidiscrimination laws (Bill S-201: Genetic Nondiscrimination Act) to avoid prejudice on the grounds of genetics (including insurance and employability) [63] (Participant 5, BC Generations Project). Several participants believed that the public acceptability of their work was important, but they “weren’t sure how to go about ensuring it” (Participant 7, Dementia Platform UK).

## Discussion

### Principal Findings

Using genomic and routinely collected data holds great potential for health research. The genomic data we identified in our literature review included SNPs, polygenic risk scores, and gene activity scores. Routine data primarily consisted of EHRs, but we did find other routine data types including registry data and deprivation indices that had been combined with genomic data. This paper shows how genomic research has progressed in recent years—from basic GWA and PheWA studies—to more complex methodologies in which health records are linked to genomic data at the individual level. Associations between genetic variants and phenotypes, identification of drug targets, knowledge about drug toxicity, and effectiveness can all be studied from the combination of genomic and routine data and leveraged for public benefit.

The EHRs created during routinely collected care provide the large sample sizes needed for GWAS analysis without the need for costly and time-consuming prospective data collection. These larger samples allow for greater validity, generalizability, and yield adequate statistical power, while also minimizing participant burden and reducing attrition during follow-up [64]. In addition, these data sets are not limited to a circumscribed number of phenotypes in the way that a traditional research study might be [40]. This richness and diversity of EHR data means that the large number of phenotypes used in PheWAS can be very clearly defined (referred to as deep phenotyping), which enhances the accuracy of phenotype-genotype associations [40].

Most importantly, with regard to EHRs, perhaps, is the additional information that routinely collected data provide about an individual’s environment. This can include lifestyle factors, education, work history, pollution, and even traumatic events. Genetic determinism has long been rejected, and we now widely accept the powerful influence that lifestyle and the environment have on the way that our genes are expressed [65-67]. Precision medicine requires novel correlations not only between genotype and phenotype to be made but also with an individual’s environment to inform new methods for diagnosing, treating, and preventing disease in a way that is tailored to that individual. Linking genomic data to routine data promises to elucidate important findings for precision medicine research, which will enable researchers to understand the relationship between an individual’s genome and their complete life course [68]. More of these types of studies are needed for precision medicine to reach its full potential, to make the intricate genotype-phenotype associations needed to advance the understanding and treatment of disease.

From our interviews, we identified 3 main ways that researchers can access genomic and routine data: publicly available on the web, released to researchers, and via a data safe haven. We also observed that biobanks and databanks seem to be moving toward renouncing a data release model and instead favoring a data safe haven approach. Aside from potentially solving data storage issues, this will also help assuage privacy and governance concerns. Reidentification and disclosure from genomic data is possible [69,70] and can lead to many undesirable consequences: discrimination by health or life insurance companies, societal stigma, and the discovery of a genetic predisposition to a condition when one does not want to be told. This is complicated further because of the familial nature of genes, and disclosure could cause some devastating effects for biological relatives as well [71-73].

Despite this, we must keep in mind that simply because genomic data are unique, this in itself does not render it identifiable. Rather, the reidentification risk of genomic data depends on the way they are accessed, the type of analyses that are conducted, and the format in which the results are finally published [70]. This means that decisions to use certain data access models based on practicality and decreased costs are not sufficient. Genomic research relies heavily on human participation, and the public should be consulted to inform the way in which their data are accessed [74]. There is a plethora of activity taking place to consult the public about health research in general, and the success and acceptability of large-scale data research is owed, in part, to the extensive public engagement activities that have been taking place [75,76]. Organizations such as the Global Alliance for Genomics and Health [77] are engaging with the public on the use of genomic data in research, but there is still more work to be done regarding the use of genomic and routinely collected data [78].

### Limitations

This review was not intended to be exhaustive or systematic; therefore, it does not include all health research studies that have used genomic and routinely collected data. We also excluded biobanks and databanks that do not house routine data; therefore, we only include examples here. This may have resulted in inadvertently excluding some countries and institutions where this research takes place and the types of genomic and routinely collected data that have been used. Not implementing systematic methods in study selection may have introduced bias in this review’s conclusions; however, the pragmatic approach used here was deemed sufficient to meet our objectives.

We included 19 studies in this review, only one of these involved data and research from a non-Western country (China) [21]. We did not come across any research that had taken place in low- to middle-income countries, although as this review was not conducted systematically, we may have unintentionally overlooked these. However, the absence of any studies from such countries may be, as Tekola-Ayele and Rotimi [79] explain, a result of having a limited number of well-trained genomic scientists and poor research infrastructure, and due to a less well-established routine data collection infrastructure and procedures such as EHRs [80].

Qualitative interviews may be subject to recruitment bias, which means that some viewpoints and experiences were excluded. In addition, there is limited information about participants; however, as this is a relatively small field of research, it was deemed necessary to maintain participants’ privacy.

### Conclusions

Given the projected increase in the availability of genomic data, the potential to be obtained from its combination with routine health data is vast. This review has shown examples of what has been done in this field so far with, for example, GWAS and PheWAS plus other study designs. For fields such as pharmacogenomics, these methodologies need to be used further, where using routinely collected data will simplify the process of tracking longer-term outcomes of personalized medical treatments, and elucidate new findings on the effects of the environment on drug-gene interactions. Our take away from this study is that there are several challenges involved in using these data, particularly surrounding privacy. Therefore, it is imperative that appropriate data governance be documented and that public engagement activities take place to ensure socially acceptable practices. Overcoming these barriers will ensure that the use of these data to progress health research can be exploited to its full potential.

## Appendix

Multimedia Appendix 1 Summary of findings.

## Abbreviations

BC: British Columbia
EHR: electronic health record
eMERGE: Electronic and Medical Records and Genomics
GAP: Genetics and Psychosis
GWAS: Genome-Wide Association Studies
ICD: International Classification of Disease
NHS: National Health Service
PheWAS: phenome-wide association studies
POND: Province of Ontario Neurodevelopmental Disorders
SAIL: Secure Anonymized Information Linkage
