# Supplementary material for: Exploring the Use of Genomic and Routinely Collected Data: Narrative Literature Review and Interview Study
Source: J Med Internet Res. 2021 Sep 24;23(9):e15739. doi: 10.2196/15739 (PMC8501405; doi:10.2196/15739)
Supplement: Multimedia Appendix 1 [file jmir_v23i9e15739_app1.docx]

Table 1 Summary of findings

| **Study** | **Country of research institution** | **No. of participants** | **Study design** | **Purpose** | **Genomic data** | **Routine data** | **Role of routine data** | **Data source** | **Data access** |
| --- | --- | --- | --- | --- | --- | --- | --- | --- | --- |
| 45 and Up [33] | Australia | ~260,000 | Longitudinal study | Identifying genomic risk of disease | SNPs | eHRs | Baseline information  Follow up | Sax Institute | Data safe haven |
| Afghahi et al [22] | USA | 10,125 | Gene expression profiling | Drugs – efficacy | Gene activity scores | eHRs  Registry records | Identifying cases and controls  Baseline information | Stanford University    Palo Alto Medical Foundation  California Cancer Registry | Released to researchers |
| Aminkeng et al [19] | Canada | 456 | GWAS | Drugs – toxicity/safety | SNPs | eHRs | Baseline information | 13 paediatric oncology centres across Canada | Released to researchers |
| Breitenstein et al [23] | USA | 111,673 | Candidate gene study | Drugs – efficacy | SNPs | eHRs | Phenotyping | Mayo Genome Consortia | Released to researchers |
| Clarke et al [15] | UK | 5,127 | GWAS | Identifying genomic risk of disease | Polygenic risk scores | Scottish Index of Multiple Deprivation | Generating variables | Generation Scotland | Released to researchers |
| Cronin et al [24] | USA | 24,198 | PheWAS | Identifying genomic risk of disease | SNPs | eHRs | Phenotyping | eMERGE  BioVU | Released to researchers |
| Crosslin et al [25] | USA | 22,981 | GWAS/PheWAS | Identifying genomic risk of disease | SNPs | eHRs | Identifying cases and controls | eMERGE | Released to researchers |
| Dementia Platform UK [16] | UK | 3,000,000 | Longitudinal study | Identifying genomic risk of disease | SNPs | eHRs | Baseline information  Follow up | Sail Databank | Data safe haven |
| Dhalla et al [31] | Canada | 30,000 | Longitudinal study | Identifying genomic risk of disease | SNPs | eHRs | Baseline information  Follow up | The Canadian Partnership for Tomorrow Project  Population Data BC | Data safe haven |
| Diogo et al [26] | USA | 697,815 | GWAS/PheWAS | Drugs – efficacy | SNPs | eHRs | Phenotyping | UK Biobank | Released to researchers |
| Hall et al [17] | UK | 43,062 | GWAS | Identifying genomic risk of disease | SNPs | eHRs | Identifying cases and controls | UK Biobank  Generation Scotland | Released to researchers |
| Hebbring et al [27] | USA | 4235 | PheWAS | Identifying genomic risk of disease | SNPs | eHRs | Phenotyping | Marshfield Clinic Personalized Medicine Research Project | Released to researchers |
| Knisely et al [28] | USA | 30 | Exploratory study | Drugs – toxicity/safety | Gene activity scores | eHRs | Baseline information | Regenstrief Institute | Released to researchers |
| Lloyd et al [18] | UK | 12,587 | Longitudinal study | Identifying genomic risk of disease | Polygenic risk scores | eHRs | Baseline information  Follow up  Phenotyping | SAIL Databank | Data safe haven |
| Millwood et al [21] | England and China | 51,217 | GWAS | Identifying genomic risk of disease | SNPs | eHRs | Follow up | China Kadoorie Biobank | Released to researchers |
| POND Network [32] | Canada | 3000 | Longitudinal study | Identifying genomic risk of disease | SNPs | eHRs | Baseline information  Follow up | IC/ES | Data safe haven |
| Rusiecki et al [29] | USA | 150 | Case control study | Identifying genomic risk of disease | DNA methylation status | eHRs | Identifying cases and controls | Armed Forces Health Surveillance Center | Released to researchers |
| Swansea Neurology Biobank [19] | UK | 400 | Case control study | Identifying genomic risk of disease | SNPs | eHRs | Follow up | SAIL Databank | Data safe haven |
| Wickramasinghe [20] | UK | 291 | Longitudinal study | Identifying genomic risk of disease | SNPs | eHRs | Follow up | The South London and Maudsley NHS Foundation Trust Biomedical Research Centre case register | Released to researchers |
